# A descriptive study of older adults with persistent pain: Use and perceived effectiveness of pain management strategies [ISRCTN11899548]

**DOI:** 10.1186/1471-2318-5-12

**Published:** 2005-11-08

**Authors:** Carol A Kemp, Mary Ersek, Judith A Turner

**Affiliations:** 1 Pain and Palliative Care Research Department, Swedish Medical Center, 500 17^th ^Ave, Providence Professional Building Suite 405, Seattle, WA 98122-5711, USA; 2 Department of Biobehavioral Nursing and Health Systems, University of Washington School of Nursing, Box 357266, Seattle, WA 98195-1406, USA; 3 Department of Psychiatry and Behavioral Sciences, University of Washington School of Medicine, Box 356560, Seattle, WA 98195-6560, USA; 4 Department of Rehabilitation Medicine, University of Washington School of Medicine, Box 356560, Seattle, WA 98195-6560, USA

## Abstract

**Background:**

Persistent pain is a common, often debilitating, problem in older adults; however, few studies have focused on the experiences of older adults in managing their pain. The objective of this study was to describe the use and perceived effectiveness of pain management strategies in a sample of older adults and to explore the associations of these variables with demographic and psychosocial characteristics.

**Methods:**

Adults ≥ 65 years old and living in retirement facilities who reported persistent pain (N = 235, mean age = 82 years, 84% female, 94% white) completed measures of demographics, pain, depression, self-efficacy for managing pain, and a Pain Management Strategies Survey. Participants identified current and previous-year use of 42 pain management strategies and rated helpfulness of each on a 5-point scale.

**Results:**

Acetaminophen, regular exercise, prayer, and heat and cold were the most frequently used pain management strategies (61%, 58%, 53%, and 48%, respectively). Strategies used by >25% of the sample that were rated moderately or more helpful (i.e., >2 on a 0 to 4 scale) were prayer [mean (SD) = 2.9 (0.9)], opioids [2.6 (0.8)], regular exercise [2.5 (1.0)], heat/cold [2.5 (1.0)], nonsteroidal anti-inflammatory drugs [2.4 (1.0)], and acetaminophen [2.3 (1.0)]. Young-old (65–74 years) study participants reported use of more strategies than did old-old (85+ years) participants (p = .03). Perceived helpfulness of strategy use was significantly associated with pain intensity (r = -.14, p < .0001), self-efficacy (r = .28, p < .0001), and depression (r = -.20, p = .003).

**Conclusion:**

On average, older adults view the strategies they use for persistent pain as only moderately helpful. The associations between perceived helpfulness and self-efficacy and depression suggest avenues of pain management that are focused less on specific treatments and more on how persons with persistent pain think about their pain.

## Background

Persistent pain is common among adults age 65 years and older [1,2], affecting 58–70% of community-dwelling older adults [3,4]. It is often associated with significant physical and psychosocial disability [5]. The most common types of persistent pain in this age group are neuropathic and musculoskeletal (e.g., low back pain, osteoarthritis pain, and pain in previous fracture sites) [2,5].

Despite the prevalence and importance of persistent pain among older adults, little research has systematically examined the pain management strategies used in this population. A few studies have examined the use of complementary and alternative therapies among people of various ages with persistent pain or diseases associated with pain, such as arthritis [6-9]. Among the studies that have examined the use of many types of conventional and complementary therapies for pain [10-14], few have focused on older adults and only three have assessed older adults' evaluations of the effectiveness of the treatments that they tried [11,12,15].

Blomqvist and Edberg [12] examined pain management strategies in a sample of 90 Swedes aged 75 years and older. Participants lived either in their own homes or in sheltered accommodations; all required assistance in activities of daily living from paid providers. The investigators used a structured interview to assess participants' use and perceived effectiveness of various pain management techniques. The most commonly used strategies were medication (used by 86% of the participants), distraction (e.g., watching television, reading, praying, meeting friends; 68%), rest (67%), and mobility (e.g., physical therapy, walking, housework; 66%). Only mobility was perceived as effective and without side effects. Medications and rest were considered effective, but associated with negative side effects, and distraction was categorized as being harmless, but having uncertain effectiveness.

Barry and colleagues [11] asked 245 predominantly male community-dwelling Veterans Affairs primary care patients aged 65–90 to describe their pain management strategies and to rate their perceived effectiveness. Analgesic medication use was the most commonly reported strategy (78%), followed by exercise (35%), cognitive coping (27%), religious activities (21%), and activity restriction (20%). Only four of the strategies were rated as quite or extremely effective by more than half of users; these strategies were "seek care of a physician" (80%), physical therapies (56%), complementary therapies (55%), and perseverance (52%). Little is known about the influence of older adults' sociodemographic and clinical characteristics on pain management strategy use and perceived effectiveness. Barry et al. [11] found that women were more likely than men to use cognitive coping methods and religious activities, and patients with persistent pain due to a musculoskeletal cause were more likely to use analgesic medications than were those with pain due to other causes. They did not find an association between age and any commonly used coping strategy. However, more studies are needed to establish whether, among adults aged 65 and older, age, gender, and other characteristics are associated with the use of specific pain management strategies or effectiveness of strategies used.

Kung et al. [15] examined the use and perceived effectiveness of pain management strategies among 230 community-dwelling Australians over age 55. The most frequently used strategies were heat (83%), distraction (82%), prescription medications (81%), rest (81%), physical exercise (79%), social activities (75%), and positive thinking (72%). The strategies perceived as most helpful among this sample were community support services, such as disability parking, home help, taxi transportation assistance, appropriate housing, and home modifications.

Depression and self-efficacy for managing pain are two characteristics that might well be associated with pain management strategy use and effectiveness. We previously reported that self-efficacy for managing pain (perceptions of personal capability to exercise control over pain or associated problems) was associated significantly and positively with use of task persistence, exercise/stretch, coping self-statements, and activity pacing to cope with pain in a sample of 140 retirement facility residents with persistent pain [16]. However, we did not examine whether self-efficacy was associated with use or perceived effectiveness of medical and complementary therapies for pain, and we could identify no studies of older adults that have addressed this question. Likewise, we could identify no studies of older adults that examined the association of depression with use or perceived effectiveness of pain management strategies. Studies of young and middle-aged adults with persistent pain have established the positive association between depression and passive pain coping (e.g., resting and guarding painful parts of the body) and the negative association between depression and active pain coping (e.g., task persistence, cognitive coping) [17-19]. Thus, it might prove fruitful to examine whether depression is associated with pain management strategy use among older adults. Furthermore, the tendency of depressed individuals to appraise experiences more negatively might result in such individuals viewing pain management strategies tried as ineffective.

Knowledge of pain management practices and perceptions of benefit is important for understanding how to support older adults in managing persistent pain. Increased understanding in this area could help clinicians better advise older patients with persistent pain as to pain management strategies most likely to be considered beneficial by the patient, and could also help identify strategies to study prospectively in more rigorous research. Given the dearth of information about persistent pain management strategies and complementary therapies used by older adults, the primary purpose of this study was to describe the use and perceived effectiveness of pain treatments, including complementary therapies, in a sample of older adults with persistent pain. In addition, we explored the associations of age, gender, pain characteristics, depression, and self-efficacy with number and perceived effectiveness of strategies used. Given the lack of previous research, we made no a priori hypotheses regarding these associations.

## Methods

### Study participants and procedures

Data for this study were collected in the baseline assessment of participants in a randomized controlled trial (RCT) evaluating the effectiveness of a pain self-management group intervention [20]. The study was approved by the institutional review board of Swedish Medical Center (Seattle, WA). All study participants provided written informed consent.

The study sample was recruited from adults residing in 43 for-profit and not-for-profit senior housing or retirement communities in the greater Seattle, Washington, area. Participants were recruited through newsletter announcements, flyers, and presentations at the communities. Although most facilities offered exclusively or predominantly independent living, some also provided assisted living. Fourteen communities offered continuing care ranging from independent living apartments to skilled nursing facilities, and eight provided subsidized housing. The majority of study participants lived independently.

Study inclusion criteria were age 65 years or older, pain of more than three months' duration that interfered with daily activities, average pain in the past week greater than 2 on a 0–10 numerical rating scale, ability to complete study questionnaires, and ability to attend seven weekly sessions at the participant's retirement facility (due to the nature of the larger RCT). Exclusion criteria were current cancer and surgery within the past six months or planned in the next six months.

**Figure 1:**
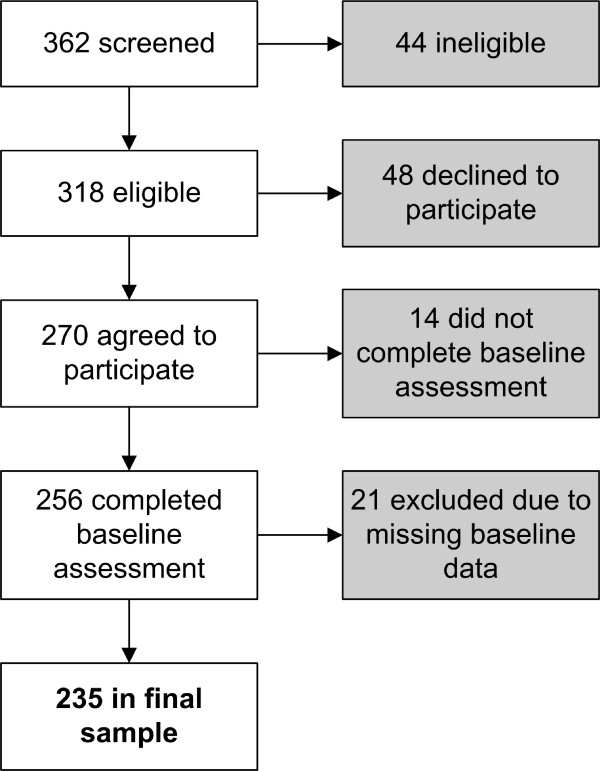
Study flow.

Among the 362 individuals screened for the study, 44 (12%) were ineligible (Figure 1). Of the 318 eligible individuals, 256 (80.5%) enrolled and completed the baseline assessment, and 62 (19.5%) declined to participate or did not complete the baseline assessment. Among the 256 study participants, 21 (8%) were not included in analyses for this report due to missing data. The 235 individuals included in the current report did not differ significantly from the 83 individuals who were eligible but declined to enroll or did not complete all of the baseline measures in age, race, income, proportion living alone, average pain in the past week, average pain interference with activity in the past week, or average pain interference with enjoyment of life in the past week. However, the sample included in the current report, as compared with those eligible but not included, had more males (16% vs. 6%, chi-square test, p = .02) and a trend toward more participants with education past high school (77% vs. 66%, chi-square test, p = .05).

### Measures

The study measures are described below. The Charlson Index of Comorbidity and the Mini-Mental State Exam were administered by study staff at the time of participant enrollment. Questions concerning pain and sociodemographic characteristics and the other measures were completed by study participants at home and were returned by mail. Because the measure used to assess pain management strategy use was developed after enrollment for the RCT began, it was completed at home and was returned by mail after the baseline assessment for 71 individuals included in this report.

#### Charlson Index of Comorbidity (CI)

The Charlson Index (CI) is an extensively used, valid, and reliable measure of comorbid conditions [21]. The CI uses 19 categories of comorbidity, which are primarily defined using *International Classification of Diseases-9-Clinical Modifications *(ICD-9-CM) diagnostic codes; each category is weighted and scored according to an algorithm. Katz et al. [22] adapted the CI for use as an interview or mailed questionnaire and found the adapted version to be reliable and valid in a group of older adults. Scores range from 0 to 35, with higher scores indicating greater health burden from comorbid causes.

#### Mini-Mental State Examination (MMSE)

The MMSE [23] is widely used to screen for cognitive impairment in older adults. It consists of 30 items and requires 5–10 minutes to administer. Items assess orientation, memory, attention, and calculation. The score is the total number of correct answers out of 30 possible; scores of 24–30 indicate no cognitive impairment, scores of 18–23 suggest mild cognitive impairment, and scores of less than 18 suggest severe cognitive impairment. The MMSE has been demonstrated to be valid and to have good test-retest reliability [24].

#### Brief Pain Inventory (BPI)

The BPI is a widely used, reliable, valid measure that assesses pain history, location, intensity, and interference with activities [25,26]. Ratings of average, current, least, and worst pain during the past week on a scale of 0 ("No pain") to 10 ("Pain as bad as you can imagine") were averaged to create a single pain intensity score [27]. Pain-related interference was calculated as the mean of 0 ("does not interfere") to 10 ("completely interferes") ratings of pain interference with general activity, mood, walking, work (including housework), relations with others, sleep, and enjoyment of life.

#### Geriatric Depression Scale (GDS)

The Geriatric Depression Scale (GDS) [28-30] is a 30-item self-report measure designed to assess depressive symptoms in older persons. Scores of 11 or higher are considered indicative of depression in older adults. Good sensitivity (84–100%) and specificity (73–96%) for detecting depression in geriatric psychiatric and medical outpatients have been demonstrated [31,32]. The GDS was selected over other available depression measures because of its screening efficiency with geriatric outpatient populations, its focus on affective rather than physical symptoms, and its true/false scoring format, which studies have found to be simpler for older adults to complete [32].

#### Self-Efficacy Scale (SES)

To assess self-efficacy for managing pain, we used the eight-item Arthritis Self-Efficacy Scale, modified by replacing the word "arthritis" with "pain" [33]. The Arthritis Self-Efficacy Scale has been demonstrated to have high internal consistency, adequate test-retest reliability, and validity [34,35]. Study participants rated on a scale from 1 = "very uncertain" to 10 = "very certain" their confidence that they can decrease their pain, keep pain from interfering with sleep, keep pain from interfering with the things they want to do, regulate activity to remain active, keep fatigue from interfering with activities, do something to feel better if they are feeling blue, manage pain during daily activities, and deal with the frustration of pain. Scores for the scale are reported as the mean of the eight ratings.

#### Pain Management Strategies Survey (PMSS)

We developed a Pain Management Strategies Survey (PMSS) to assess the use and perceived effectiveness of 42 medical, complementary, and self-care strategies used by older adults to manage persistent pain (see Additional file 1). The instrument was adapted from the work of Warms, Turner, Marshall, and Cardenas [13]. Several items were added to capture complementary therapies that were not included in the Warms et al. instrument [36].

Space was provided to allow respondents to add up to four therapies beyond those listed. Study participants were asked to indicate whether they were using each strategy currently or had used it in the past year, and if so, to rate the strategy's helpfulness on a scale of 0 = "not at all helpful" to 4 = "extremely helpful." A value of 2 was labeled as "moderately helpful."

### Statistical analysis

Each pain management strategy was analyzed as a dichotomous variable ("used" versus "not used" currently and/or in the past year). We used descriptive statistics to summarize the demographic characteristics of the sample, the strategies used, and their perceived effectiveness. To examine age differences, we categorized age into young-old (65–74 years), mid-old (75–84 years), and old-old (85 or more years). We inspected the distributions of number of pain sites; scores on the SES, GDS, pain intensity, and pain interference measures; strategy helpfulness scores for strategies used by 25% or more participants (to ensure an adequate subgroup size); and number of treatments used; none were substantially skewed (i.e., skewness > 1.0). For each participant, ratings of helpfulness of strategies used currently or in the past year were averaged to create a single mean strategy helpfulness score. We used *t*-tests to examine gender differences in number of treatments used and analyses of variance (ANOVA) to examine whether the age groups differed in number of treatments used and perceived helpfulness. We used Pearson's correlation and chi-square analyses to examine associations between participant strategy use/perceived effectiveness and age, gender, pain characteristics, depression, and self-efficacy. Finally, we conducted sensitivity analyses to determine if there were significant differences in results for the 71 participants who completed the PMSS after the baseline assessment versus the other study participants. We considered the most important comparison to be in terms of proportions of participants who endorsed the use of the strategies taught in the self-management classes, because it is possible that if the survey was completed after beginning the intervention, survey responses could be affected by intervention content. We conducted chi-square analyses on the use of relaxation, exercise, heat/cold, opioids, NSAIDS, acetaminophen, anti-seizure medications, and antidepressants between the two groups. Analyses were conducted using SPSS for Windows software, version 11.5 (Chicago, IL).

## Results

### Sample characteristics

The sample (N = 235) was 84% female and 94% white. Seventy-seven percent were educated beyond high school and 72% lived alone (Table 1). The mean (SD) age was 82 (6.3) years (range 65–99 years). The mean CI score was 1.2 (SD = 1.4, range 0–30) and 91% of the sample scored 3 or less, indicating a relatively healthy sample. On average, the sample reported moderate pain intensity [mean (SD) = 5.4 (1.8)] and pain-related interference [mean (SD) = 4.3 (2.0)] on the BPI. The mean (SD) scores for the Geriatric Depression Scale and the Self-Efficacy Scale were 8.4 (5.7) and 5.6 (1.9), respectively. Three participants (1%) had an MMSE score under 24 (MMSE data were not available for 10 participants). Eighty-five percent of the study participants reported pain in more than one location [mean (SD) = 3 (1.5)] (Table 2). Seventy-four percent reported pain in the lower extremities, 57% reported pain in the back, and 55% reported pain in the buttocks/hips.

**Table 1:** Sample demographic characteristics (n = 235)

**Characteristic**	**Age 65–74**	**Age 75–84**	**Age 85+**	**Total n**	**(%) of Sample**
**Gender**					
Male	5	24	9	38	(16)
Female	30	90	77	197	(84)
**Living arrangement**					
Lives alone	24	78	67	169	(72)
Lives with someone	11	35	19	65	(28)
Not reported				1	(<1)
**Education**					
High school or less	10	22	21	53	(23)
Post-secondary education	25	91	64	180	(77)
Not reported				2	(<1)
**Income (annual)**					
<$45,000	30	72	56	158	(67)
$45,000 or more	2	30	10	42	(18)
Not reported				35	(15)
**Ethnicity**					
Hispanic or Latino	1	3	3	7	(3)
Not Hispanic or Latino	34	109	83	226	(96)
Not reported				2	(1)
**Race**					
White	29	106	85	220	(94)
Non-white	6	8	1	15	(6)

**Table 2:** Pain locations by age group (n = 235)

**Pain Locations**	**Age 65–74 (%)**	**Age 75–84 (%)**	**Age 85+ (%)**	**Total n**	**(%) of Sample**
Legs or feet	29 (17)	84 (49)	60 (35)	173	(73.6)
Back	24 (18)	63 (47)	47 (35)	134	(57.0)
Buttocks or hip	21 (16)	64 (50)	44 (34)	129	(54.9)
Shoulders	16 (16)	44 (44)	41 (41)	101	(43.0)
Arms or hands	14 (17)	39 (48)	28 (35)	81	(34.5)
Neck	11 (22)	23 (46)	16 (32)	50	(21.3)
Head	4 (22)	9 (50)	5 (28)	18	(7.7)
Chest	3 (20)	8 (53)	4 (27)	15	(6.4)
Abdomen	1 (9)	7 (64)	3 (27)	11	(4.7)

### Pain management strategy use and perceived helpfulness

The pain management strategies used most frequently were acetaminophen (used by 61% of the sample), regular exercise (58%), prayer (53%), and heat or cold (48%) (Table 3). Eighty percent were currently using or had used in the previous year at least one analgesic or adjuvant medication; 36% used two or more. The mean (SD) number of strategies reported was 5.6 (3.2) (range = 0–20). The 71 participants who completed the PMSS after the baseline assessment were significantly more likely to use relaxation (48% vs. 10%, χ^2 ^= 41.05, p = .0001) or antidepressants (18% vs. 9%, χ^2 ^= 4.65, p = .03) than exercise, opioids, acetaminophen, NSAIDS, heat/cold, antiseizure medications or antidepressants as compared with the participants who completed the PMSS as part of the baseline assessment.

**Table 3:** Treatments used by study participants (n = 235) and perceived helpfulness

**Strategy**	Reported use*		Helpfulness**		Rated strategy > moderately helpful %***
			
	**n**	**%**	**Mean**	**(SD)**	
Acetaminophen (e.g., Tylenol^®^)	143	61	2.3	(1.0)	40
Regular exercise program (e.g., walking, swimming, lifting weights)	136	58	2.5	(1.0)	43
Prayer or spiritual practice	124	53	2.9	(0.9)	59
Heat or cold	112	48	2.5	(1.0)	45
Glucosamine &/or chondroiton	96	41	1.7	(1.2)	20
Physical therapy	88	37	2.0	(1.2)	30
Creams or ointments (e.g., Icy Hot^®^, Tiger Balm^®^, capsaicin)	73	31	1.9	(0.9)	21
NSAIDS (e.g., Motrin^®^, Celebrex^®^)	60	26	2.4	(1.0)	48
Opioids (e.g., Vicodin^®^, Tylenol^® ^#3, morphine)	59	25	2.6	(0.8)	52
Relaxation techniques (e.g., meditation, relaxation response, progressive muscle relaxation)	51	22	2.1	(0.9)	24
Injection of medication directly into joint (e.g., knee, hip)	34	15	2.2	(1.7)	58
Massage therapies (e.g., Rolfing, Swedish, shiatsu)	31	13	2.2	(1.1)	36
Antidepressants (e.g., nortriptyline, desipramine)	27	12	2.2	(1.1)	42
Chiropractic care	26	11	2.3	(1.6)	44
Anti-seizure medications (e.g., Neurontin^®^)	25	11	2.1	(1.5)	36
High-dose or mega-vitamin therapies, not including a daily vitamin or vitamins prescribed by your physician	20	9	1.4	(1.3)	18
Splints or braces	19	8	2.3	(0.9)	42
Special diet programs (or losing or gaining weight, like the kind you have to pay for, but not including trying to lose or gain weight on your own)	19	8	1.7	(1.5)	29
Spiritual or religious healing by others	17	7	3.0	(0.8)	69
Acupuncture	17	7	1.1	(1.2)	7
Energy healing (e.g., magnets, energy machines, the laying of hands, Reiki, Therapeutic Touch)	16	7	1.5	(1.0)	13
Movement therapy (e.g., yoga, tai chi, feldenkrais)	15	6	2.4	(1.5)	53
Foot reflexology	14	6	1.7	(1.2)	23
Chronic illness or arthritis education classes	10	4	2.0	(0.8)	25
Herbal therapies (e.g., arnica, evening primrose)	10	4	2.0	(1.5)	33
TENS unit	9	4	1.4	(1.6)	22
A lifestyle diet like vegetarianism or macrobiotics	8	3	2.5	(1.2)	63
Special jewelry (e.g., copper bracelet)	8	3	0.4	(0.8)	0
A self-help group, other than this study	7	3	2.4	(1.1)	57
Imagery techniques (e.g., guided imagery)	7	3	1.6	(1.1)	14
Homeopathy	6	3	1.5	(1.8)	33
Lidoderm patch	6	3	2.4	(1.8)	60
Infusion of pain medication directly into spine using a pump	4	2	2.3	(2.1)	50
Spinal cord stimulator	4	2	2.5	(1.3)	50
Folk remedy	3	1	3.0	(1.0)	67
Naturopathy	3	1	1.3	(1.2)	0
Osteopathy	3	1	4.0	(0.0)	100
Psychotherapy/counselling	3	1	2.3	(1.5)	33
Aromatherapy	3	1	1.5	(0.7)	0
Biofeedback	2	1	1.0	(1.4)	0
Hypnosis	2	1	3.0	(1.4)	50
Nerve blocks	2	1	3.5	(0.7)	100

NSAIDS – non-steroidal anti-inflammatory medications; TENS – transcutaneous electrical nerve stimulation

*Currently or in past year

**Scale = 0–4

*** Among participants who reported use of the strategy, percent who rated it as more than moderately helpful (3 or 4 on 0–4 scale).

Participants wrote in 46 responses in the "other" category. Of these, 27 were redundant with strategies listed on the survey. If the respondent did not indicate the use of that strategy as listed on the survey, we considered the write-in response to be the same a checking that strategy on the PMSS; these responses were included in the results shown in Table 3. The remaining 19 strategies not listed on the survey, but written in by participants, were rest, reading, music, elevate feet, elastic stockings (each written in by two participants), and "shoe inserts," "walk with walker," "considering surgery," "therapeutic mattress," "foot soaks," "brain/mind," "stop reading in bed," "nighttime snacks," and "alcohol" (each written in by one participant).

We limited our examination of ratings of helpfulness of strategies to the strategies endorsed by 25% or more of the sample, in order to ensure a sufficient size in subgroup analyses. Mean helpfulness ratings ranged from 1.7 (glucosamine) to 2.9 (prayer). Table 3 also shows the percentages of participants who rated the helpfulness of a treatment used as a 3 or 4 (extremely helpful). Seventy-four percent of the sample rated at least one strategy as a 3 or 4. Among the strategies, only prayer, opioid medication, and joint injections were rated 3 or 4 by more than 50% of participants who used the strategy.

### Gender and age differences in strategy use and perceived helpfulness

Among the comparisons of men versus women in the use of each of the strategies reported by 25% more of the sample only one statistically significant difference emerged. Women were more likely than men to report use of heat or cold (51% vs. 29%, chi-square test, p = .01). Men and women did not differ significantly in mean treatment helpfulness ratings or number of treatments tried.

The three age groups differed significantly in number of treatments used (ANOVA, p = .03). Post-hoc contrasts (Tukey HSD) revealed that those aged 65–74 used more strategies on average [mean (SD) = 6.9 (3.5)] than did those aged 85 or older [mean (SD) = 5.3 (2.9); p = .03]. There was a trend toward the use of more strategies by those aged 65–74 than by those aged 75–84 [mean (SD) = 5.5 (3.3); p = .05]. There were no statistically significant differences by age group in the use of each of the 10 strategies reported most frequently, although there was a trend toward a significant difference in the use of relaxation (reported by 34% of those aged 65–74 years, 16% of those aged 75–84 years, and 24% of those aged 85 years or older; chi-square test, p = .05). The three age groups did not differ significantly in mean treatment helpfulness ratings.

### Association of pain, depression, and self-efficacy with number of pain management strategies used and helpfulness

Number of strategies used was not associated significantly with pain intensity, pain interference, depression, or self-efficacy scores, but was associated positively with number of body pain locations (r = .26, p < .0001). Mean treatment helpfulness scores were associated negatively with pain intensity (r = -.14, p = .02), pain interference (r = -.18, p = .006) and depression (r = -.20, p = .003), and positively with self-efficacy scores (r = .28, p < .0001). There was no significant association between mean helpfulness and number of painful body locations.

## Discussion

In this sample of older adults with persistent pain, most participants reported use of multiple pain management strategies that were perceived as only moderately effective on average. However, 74% rated at least one strategy as a 3 or 4 on the 0–4 (4 = "extremely helpful") helpfulness scale, indicating that the majority of the sample found at least one strategy that was more than moderately effective for their pain. The most commonly used strategies that were assessed as being the most effective by users included prayer or spiritual practice, opioids, nonsteroidal anti-inflammatory drugs, heat and cold, and physical exercise. We found little difference between genders or age groups within our sample of older adults in pain management strategies used.

In this sample, "prayer or spiritual practice" was the third most commonly reported strategy (with only acetaminophen and regular exercise reported by more participants). Among strategies endorsed by at least 25% of participants, this strategy was rated as most helpful on average. Barry et al. [11] also found that religious activities were one of the most commonly reported pain coping strategies in a sample of older adults, and almost half of their sample rated this strategy as quite or extremely effective. Dunn and Horgas [37] found that among older adults with religious affiliations, women and racial minorities were more likely to report using religious coping strategies to manage pain. These findings suggest the need for further study of prayer and religious practices as used by older adults to cope with persistent pain, and the potential value for clinicians to inquire about this in understanding how their patients manage pain.

Older adults appear to rely in large part on medications to manage persistent pain. In this sample, 80% currently used or had used in the past year at least one analgesic or adjuvant medication; 36% used two or more. The high reported use of analgesics among older adults with pain is consistent with previous studies [11,12,15,38]. Moreover, the perceived effectiveness for some analgesics was relatively high. These findings indicate that analgesic therapy can be helpful in this group; however, additional studies are necessary to explore the effectiveness of medications while taking into account the risk of adverse effects.

Only slightly more than half of the sample reported use in the past year of a regular exercise program to manage pain. This percentage is considerably higher than that reported by Barry et al. [11] (35% reported using the strategy in the past month), but lower than reported by Kung et al. [15] (79% of their sample used exercise as a pain management strategy). In all three studies, exercise was rated as quite/extremely helpful/effective by 26–43% of older adults who used the strategy to manage pain. The reason for the variation in perceived effectiveness is unclear, but may indicate the need for more structured exercise programs that target pain and inclusion of activity pacing into patient teaching about persistent pain. Several studies have documented the benefits of structured exercise programs in decreasing pain [39-42] in older adults; our findings suggest the need for more research on interventions to increase regular exercise among older adults with persistent pain.

Significantly more participants in the group who completed the PMSS after baseline data collection used relaxation and antidepressant medications as pain management strategies. Relaxation strategies were covered in weeks two and three of the seven-week self-management program (for participants randomized to the program), which may have influenced the reported use of relaxation by participants who completed the PMSS after beginning the self-management program. Antidepressants were covered in week six of the classes, making the intervention influence less likely for this strategy; this difference may be due to chance.

In general, the characteristics of study participants that we examined were not associated with number or type of strategies used, although women, the young-old, and those with pain in multiple locations tended to report use of more strategies. Keefe et al. reported few differences in coping strategies between men and women with osteoarthritis pain, although women used more problem-focused coping than men [43]. Barry et al. [11] found that women were more likely than men to use prayer to cope with their pain; in our study women were more likely to use hot and cold than men, but there were no significant differences in the use of prayer. It is unclear how much the differences in samples (i.e., younger age in Keefe et al.'s sample, high percentage of men in Barry et al.'s study, high percentage of women in the current study) influenced the findings related to gender. To our knowledge, this is the first study to observe a difference among different age groups over age 65 in number of pain management strategies used; further research is needed to confirm this finding in other samples and to determine reasons for differential use in this group. A possible explanation for the association between number of pain sites and number of strategies used is the use of different pain therapies for different pain problems.

Not surprisingly, study participants who reported greater pain intensity and depressive symptom severity viewed pain treatments tried as less effective, and study participants with greater self-efficacy for managing their pain reported treatments as more effective. In this exploratory study, we did not construct multivariate models to examine how pain intensity, depression, and self-efficacy interacted in explaining variance in treatment effectiveness ratings. The bivariate findings could help guide future studies constructed to test hypotheses concerning relative contributions of these variables to perceptions of treatment effectiveness. Despite the limitations of bivariate analyses, the findings suggest the potential value of interventions to treat depression and increase sense of self-efficacy for managing pain for older adults with persistent pain. It is possible that medical and complementary pain treatments might be more beneficial when patients are less depressed and have more confidence in their ability to manage their pain.

Several study limitations need to be acknowledged. First, given the multiple comparisons conducted, some significant associations may have been found by chance. We elected not to adjust for multiple comparisons given the exploratory nature of this descriptive study; further research is needed to replicate the associations found in other samples. Second, recall biases and inaccuracies may have affected the reports of treatments used and their helpfulness. Participants may have misinterpreted items on the PMSS, resulting in inaccurate reporting. For example, participants may not have known whether they used treatments such as homeopathy, glucosamine, "folk remedy," and imagery. The perception of a treatment as helpful may be due to reasons other than active ingredients of the therapy, such as placebo effects and natural history. Third, several factors (such as treatment intensity, duration, and adherence) that may have affected outcomes were not assessed. Fourth, participants in this study chose to enroll in an RCT of a self-management program for persistent pain and thus may have differed from older adults with pain not interested in participating in such a study, resulting in sample bias. The generalizability of our findings to older adults with different sociodemographic characteristics is unknown. Finally, the study results should not be interpreted as evidence for or against the effectiveness of specific treatments or pain self-management strategies. Better evidence for or against efficacy will come from high-quality RCTs.

## Conclusion

Despite these limitations, the study findings indicate that as a group, older adults appear willing to try a variety of strategies to help manage persistent pain. Gender and age do not appear to influence which strategies are tried. The findings point to the need for further research in several areas: (1) to learn more about the use of prayer and spiritual practices by older adults to manage persistent pain, (2) to develop interventions effective in increasing the use of regular exercise among older adults with persistent pain, and (3) to explore further the relationships among depression, pain intensity, self-efficacy, and pain management strategy use and perceived effectiveness. Interventions to increase self-efficacy for managing pain and decrease depression in this population may be helpful in improving pain and response to medical and complementary therapies.

## Competing interests

The author(s) declare that they have no competing interests.

## Authors' contributions

All authors contributed to the design of the study, specification of study questions addressed, and decisions regarding statistical analyses to address these questions. All authors read and approved the final manuscript.

## Pre-publication history

The pre-publication history for this paper can be accessed here:



## Supplementary Material

Additional File 1 PMSS questionnaire. Click here for file
