# Supplementary material for: A descriptive study of older adults with persistent pain: Use and perceived effectiveness of pain management strategies [ISRCTN11899548]
Source: BMC Geriatr. 2005 Nov 8;5:12. doi: 10.1186/1471-2318-5-12 (PMC1298294; doi:10.1186/1471-2318-5-12)
Supplement: Additional File 1 — PMSS questionnaire. [file 1471-2318-5-12-S1.pdf]

Pain Management Strategies Survey

We are interested in learning how older adults treat ***chronic pain***. For each of the therapies below, please answer the following questions with regard to your treatment of your ***chronic pain***, by filling in the appropriate bubbles.

1. In the past year, have you used this therapy to treat your chronic pain? (Fill bubble if **yes**.)
2. Are you currently using this therapy to treat your chronic pain? (Fill bubble if **yes**.)
3. How helpful did you find this therapy in managing your chronic pain? (Rank on a scale of 0–4, where ***0 = not at all helpful***, and ***4 = extremely helpful***. Leave blank if you have never used this therapy.)

TIMEPOINT 1

___	___	___
0	0	0
1	1	1
2	2	2
3	3	3
4	4	4
5	5	5
6	6	6
7	7	7
8	8	8
9	9	9

Therapy	Used in past year	Currently using	Helpfulness in managing pain<br>*not at all helpful*		*moderately helpful*		*extremely helpful*
1. Acupuncture	○	○	0	1	2	3	4
2. Chiropractic care	○	○	0	1	2	3	4
3. Homeopathy	○	○	0	1	2	3	4
4. Glucosamine &/or chondroitin	○	○	0	1	2	3	4
5. Herbal therapies (e.g., arnica, evening primrose)	○	○	0	1	2	3	4
6. High-dose or mega-vitamin therapies, not including a daily vitamin or vitamins prescribed by your physician	○	○	0	1	2	3	4
7. Prayer or spiritual practice	○	○	0	1	2	3	4
8. Spiritual or religious healing by others	○	○	0	1	2	3	4

Therapy	Used in past year	Currently using	Helpfulness in managing pain<br>*not at all helpful*		*moderately helpful*		*extremely helpful*
9. Special diet programs for losing or gaining weight, like the kind you have to pay for, but not including trying to lose or gain weight on your own	◯	◯	0	1	2	3	4
10. A lifestyle diet like vegetarianism or macrobiotics	◯	◯	0	1	2	3	4
11. A folk remedy of any kind (describe:_______________________)	◯	◯	0	1	2	3	4
12. A self-help group, other than this study	◯	◯	0	1	2	3	4
13. Foot reflexology	◯	◯	0	1	2	3	4
14. Relaxation techniques (e.g., meditation, relaxation response, progressive muscle relaxation)	◯	◯	0	1	2	3	4
15. Imagery techniques (e.g., guided imagery)	◯	◯	0	1	2	3	4
16. Massage therapies (e.g., Rolfing, Swedish, shiatsu)	◯	◯	0	1	2	3	4
17. Energy healing (e.g., magnets, energy machines, the laying on of hands, Reiki, Therapeutic Touch)	◯	◯	0	1	2	3	4
18. Biofeedback	◯	◯	0	1	2	3	4
19. Hypnosis	◯	◯	0	1	2	3	4
20. Naturopathy	◯	◯	0	1	2	3	4

Therapy	Used in past year	Currently using	Helpfulness in managing pain				
			not at all helpful		*moderately helpful*		*extremely helpful*
21. Osteopathy	◯	◯	⓪	①	②	③	④
22. Aromatherapy	◯	◯	⓪	①	②	③	④
23. Physical Therapy	◯	◯	⓪	①	②	③	④
24. Regular exercise program (e.g., walking, swimming, weight lifting)	◯	◯	⓪	①	②	③	④
25. Movement therapy (e.g., yoga, tai chi, feldenkrais)	◯	◯	⓪	①	②	③	④
26. Creams or ointments (e.g., Icy Hot®, Tiger Balm®, capsaicin)	◯	◯	⓪	①	②	③	④
27. NSAIDs (e.g., Motrin®, Celebrex®)	◯	◯	⓪	①	②	③	④
28. Acetaminophen (e.g., Tylenol®)	◯	◯	⓪	①	②	③	④
29. Opioids (e.g., Vicodin®, Tylenol® #3, morphine)	◯	◯	⓪	①	②	③	④
30. Anti-seizure medications (e.g., Neurontin®)	◯	◯	⓪	①	②	③	④
31. Antidepressants (e.g., nortriptyline, desipramine)	◯	◯	⓪	①	②	③	④
32. Nerve blocks	◯	◯	⓪	①	②	③	④
33. Pain medication delivered by a patch on the skin (specify:___________________)	◯	◯	⓪	①	②	③	④
34. Infusion of pain medication directly into spine using a pump	◯	◯	⓪	①	②	③	④

Therapy	Used in past year	Currently using	Helpfulness in managing pain<br>*not at all helpful*		*moderately helpful*		*extremely helpful*
35. Injection of medication directly into joint (e.g., knee, hip)	◯	◯	⓪	①	②	③	④
36. Psychotherapy/counseling	◯	◯	⓪	①	②	③	④
37. Spinal cord stimulator	◯	◯	⓪	①	②	③	④
38. TENS unit	◯	◯	⓪	①	②	③	④
39. Heat or cold	◯	◯	⓪	①	②	③	④
40. Splints or braces	◯	◯	⓪	①	②	③	④
41. Chronic illness or arthritis education classes	◯	◯	⓪	①	②	③	④
42. Special jewelry (e.g., copper bracelet)	◯	◯	⓪	①	②	③	④
43. Other therapies, not listed above, to treat your chronic pain:							
A) ______________________________	◯	◯	⓪	①	②	③	④
B) ______________________________	◯	◯	⓪	①	②	③	④
C) ______________________________	◯	◯	⓪	①	②	③	④
D) ______________________________	◯	◯	⓪	①	②	③	④
